# On the Room-Temperature Creep Behavior and Its Correlation with Length Scale of a LiTaO_3_ Single Crystal by Spherical Nanoindentation

**DOI:** 10.3390/ma12244213

**Published:** 2019-12-15

**Authors:** Wei Hang, Xianwei Huang, Min Liu, Yi Ma

**Affiliations:** 1 College of Mechanical Engineering, Zhejiang University of Technology, Hangzhou 310014, China; whang@zjut.edu.cn (W.H.); huangxw@zjut.edu.cn (X.H.); lium@zjut.edu.cn (M.L.); 2 Key Laboratory of Special Purpose Equipment and Advanced Manufacturing Technology Ministry of Education, Zhejiang University of Technology, Hangzhou 310014, China

**Keywords:** lithium tantalite, nanoindentation, creep, size effect, strain rate sensitivity

## Abstract

Relying on nanoindentation technology, the room-temperature creep behavior of a LiTaO_3_ single crystal in the typical orientation (01 $\bar{1}$ 2), i.e., Y-42° plane was investigated. Three kinds of spherical tips with the radii of 0.76, 2.95 and 9.8 μm were respectively applied to detect nanoindentation length scale effect on creep deformation at both elastic and plastic regions. Superficially, both creep displacement and rate were nearly linearly increased with increasing holding depth and independent of tip size, which could be ascribed to the simultaneously enlarged holding strain and deformation volume beneath the indenter. At a similar holding strain, creep deformation, i.e., creep strain and strain rate were more pronounced under smaller spherical tips. Strain rate sensitivities of creep flows under different spherical tips and holding strains were also estimated. The potential room-temperature creep mechanism of LiTaO_3_ under high shear compression stress was discussed.

## 1. Introduction

As a typical multi-functional material, Lithium tantalite (LiTaO_3_ or LT) exhibits excellent and unique characteristics, e.g., optical, piezoelectric and ferroelectric properties. These properties make LiTaO_3_ well-suited to numerous applications, including photoelectron devices, surface acoustic wave (SAW) substrates, pyroelectric infrared detectors, etc. [1,2]. From the perspective of deformation manner, LiTaO_3_ is classified as a typical kind of soft-brittle material with low fracture toughness of about 0.39 MPa·m^1/2^ [3], which is only one-third that of silicon, or one-tenth that of sapphire [4]. Accordingly, cracks are prone to generate on LiTaO_3_ surface, inducing catastrophic fracture during precision machining like grinding, lapping or polishing. In comparison to its bulk counterpart, crack nucleation of ultrathin LiTaO_3_ could be more sensitive to surface defects such as tiny scratches, nano-voids and surface/sub-surface damage layers. Therefore, the risk of catastrophic breakage of LiTaO_3_ would be higher when decreasing its thickness by machining to meet application requirements. In recent years, micro/nano mechanical investigations on the surface of thin LiTaO_3_ single crystals have been at the cutting edge in the use of nanoindentation technology. Gruber et al. studied the characteristics of nanoindentation morphologies in LiTaO_3_ under Berkovich and spherical indenters [5,6]. Ma et al. investigated the orientation effect on the incipient plasticity and yield stress of LiTaO_3_ by spherical nanoindentation [7]. In addition, Gruber et al. also reported the effect of surface flaws on the strength and its statistical distribution in LiTaO_3_ [8]. On the other hand, the LiTaO_3_ surface suffers long-term extrinsic pressure stresses due to abrasive particles during the machining process. In some cases, the LiTaO_3_ substrate could bear high local temperature and elastic stress when serving as an electrical device. However, the time-dependent plastic deformation of LiTaO_3_ has rarely been studied hitherto.

In comparison to conventional creep measurements, nanoindentation has merits in that it is convenient, time-saving, and accurate, particularly for small-dimension and brittle materials [9,10]. Notwithstanding the unavoidable influence of thermal drift, which greatly hinders high-temperature mechanical investigations, nanoindentation has been widely adopted to explore the time-dependent plastic deformation of materials at room temperature [11,12,13,14,15]. In the authors’ previous work [16], the room-temperature creep behavior and its correlation with structure orientation of a LiTaO_3_ single crystal were revealed by nanoindentation with a standard Berkovich indenter. However, the detailed creep deformations concerned with holding stress, strain and nanoindentation length scale were easily missing under the self-similar Berkovich indenter. In recent years, the effect of nanoindentation size on mechanical properties has attracted lots of attention [17,18,19]. With this in mind, we aim to unfold further characteristics of creep behavior of LiTaO_3_ by spherical tips. For spherical nanoindentation, the deformation region beneath indenter gradually expands from elastic to plastic deformation and the continuous variations of both stress and strain could be attained before the holding stage. In the current work, three spherical tips with radii of 0.76, 2.95 and 9.8 μm were adopted, and a vast range of holding loads was performed for each indenter in the typical orientation (01 $\bar{1}$ 2), i.e., Y-42° plane of LiTaO_3_. The room-temperature creep behavior and its correlation with nanoindentation length scale were systematically investigated at the nano and micro scales. Importantly, nanoindentation creep deformation under spherical tip could be comparable to that under high-stress uniaxial holding at room temperature. The effects of holding strain and stress state, i.e., elasticity and plasticity, on the creep deformation under each spherical tip were studied. Meanwhile, the sample size effect on the time-dependent plastic deformation in brittle LiTaO_3_ could be revealed under a similar holding strain. To illustrate the creep mechanism of the LiTaO_3_ single crystal, the strain rate sensitivity of creep flow was also estimated.

## 2. Materials and Methods

**Figure 1:**
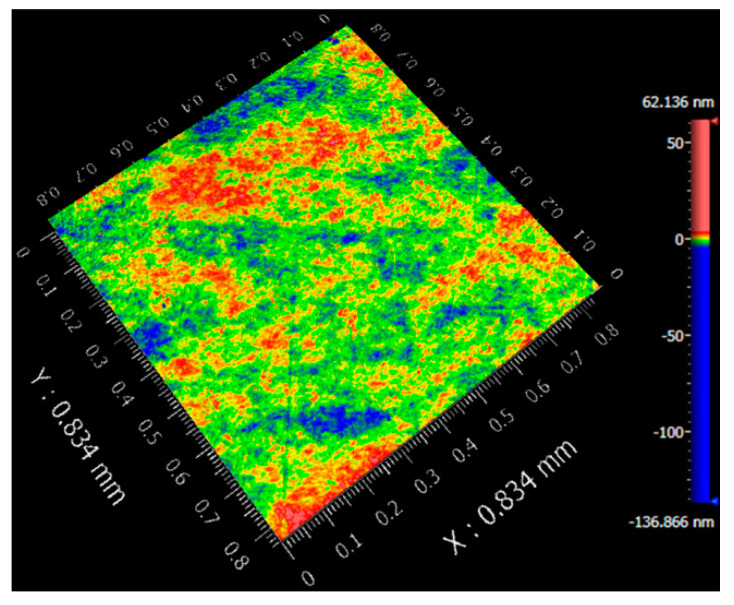
Plane surface morphologies on the area of 0.834 × 0.834 mm^2^ by optical profiler.

Prior to nanoindentation, the Y-42° plane of a commercial LiTaO_3_ single crystal wafer was carefully polished to a mirror surface. The surface morphology was detected by optical profiler (Newview 800, Zygo, Middlefield, CT, USA), as exhibited in Figure 1. Surface defects like tiny scratches or embedment of abrasives could not be observed on the polished surface. In addition, the surface roughness was about 1.1 nm on the area of 0.832 × 0.832 mm^2^. Nanoindentation load-holding tests were performed on Agilent Nano Indenter G200 (USA) at an ambient temperature of 23 °C by air conditioning. All the mechanical measurements were compliant with ISO 14577. The effective spherical tip radius was estimated by calibrating the standard fused silica. The loading rate and holding time were fixed to 2 mN/s and 500 s, respectively. The peak loads ranged from 1 to 20 mN for 0.76 μm tip, 2.5 to 50 mN for 2.95 μm tip and 25 to 450 mN for 9.8 μm tips, respectively. To minimize the thermal drift influence, all the holding tests were carried out until thermal drift decreased to 0.03 nm/s. Meanwhile, drift correction which was calibrated at 10% of the maximum load during the unloading process, was strictly performed. To ensure the reliability of the creep results, more than eighteen nanoindentation measurements were conducted for each test.

## 3. Results and Discussion

**Figure 2:**
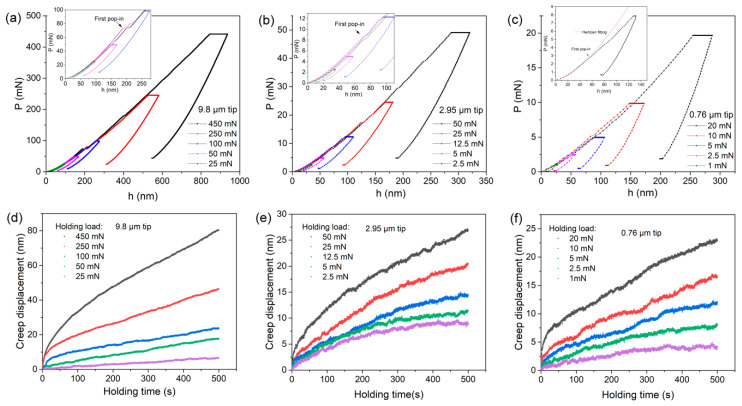
Typical load versus displacement (*P-h*) curves at various peak loads for creep tests under spherical tips with radii of (**a**) 9.8 μm, (**b**) 2.95 μm and (**c**) 0.76 μm. In addition, the corresponding creep displacements during the holding stage at various holding loads for the three spherical tips with radii of (**d**) 9.8 μm, (**e**) 2.95 μm and (**f**) 0.76 μm.

Figure 2a–c exhibits the representative load versus displacement (*P-h*) curves of load-holding tests under various spherical tips. During the loading sequences, pop-in events which could be regarded as incipient dislocation move and/or micro crack initiation were clearly observed under spherical tips of 9.8 and 2.95 μm radii, as indicated in the insets. For the spherical tip of 0.76 μm radius, the missing pop-in event could be due to the low sampling frequency (5 Hertz). By performing individual nanoindentation tests with a higher data acquisition rate (15 Hertz), as shown in the inset of Figure 2c, the occurrence of incipient plasticity under the minimum spherical tip could also be discernable and confirmed by Hertzian elastic theory [20,21,22]. In brittle materials, micro-/nano-flaws always play a more important role on the fracture behavior compared with ductile materials, and the fracture strength falls in a wide range, also known as the Weibull distribution [23]. In another work being prepared by the authors, the scattering distribution of incipient plasticity on the surface of LiTaO_3_ single crystal was revealed under the same spherical tips, as shown in Figure S1 in the Supplementary Materials. The mean loads at the first pop-in event in the Y-42° plane were 59.3 ± 16.3, 9.5 ± 2.1 and 3.9 ± 1.2 mN with spherical tips with radii of 9.8, 2.95 and 0.76 μm, respectively. Accordingly, the two minimum holding loads, i.e., 25 and 50 mN for the 9.8 μm tip, 2.5 and 5 mN for the 2.95 μm tip, 1 and 2.5 mN for the 0.76 μm tip, could be regarded as elastic holdings. In addition, the other holding tests were conducted in plastic regions. Clearly, irreversible time-dependent deformation, i.e., creep, occurred at the holding stage for all the tips, even at small holding loads.

Figure 2d–f exhibits the representative correlations between creep displacement and time at the holding stages. To compare creep deformations at various holding loads more clearly, the onsets of holding displacement and time were set to be zero. Creep flows, i.e., creep displacement and rate, were evidently increased with increasing holding loads under all the spherical tips. In the light of traditional creep behavior by uniaxial testing, three typical creep stages successively appeared, namely, primary creep (stage 1), steady-state creep (stage 2), and tertiary creep (stage 3), which could be distinguished by creep strain rate, leading to fracture. In comparison, stage 3 and fracture are unable to occur at nanoindentation creep deformation for the shear-compression stress and gradually involved fresh volume beneath indenter. Additionally, the primary creep stage, in which the creep strain rate rapidly changes with time or displacement, was much more transient due to spherical nanoindentation. The primary creep was limited to within about 50 s in plastic holdings, and was even missing in elastic holdings in LiTaO_3_. The steady-state stage, in which creep displacement increased almost linearly with holding time, occupied the vast majority of creep flow under spherical indenters.

The apparent creep deformations of LiTaO_3_ under spherical tips could be explained from the perspective of the plastic mechanism. For the elastic holding test, it was impossible for atomic diffusion to occur between the indenter and surface of LiTaO_3_ at room temperature. In addition, dislocation movement could not be the main deformation carrier in this brittle ceramic, although pre-existing dislocations or twins might be introduced in the damage layer by grinding. Therefore, it was difficult for creep deformation to occur in the region suffering elastic deformation, particularly for high-melting materials, by conventional holding test at room-temperature. However, creep deformation in elastic holdings could not be ignored in the present case, although it was subtle. The non-uniform stress distribution under spherical tip was suggested to be the intrinsic reason for the occurrence of creep flow in elastic holdings. Despite the holding loads being below the position of first pop-in event, the maximum shear stress beneath the indenter could be beyond the yield stress [14]. A critical plastic zone space is required to generate incipient dislocation or crack [24]. Accordingly, the high local stress under elastic holdings agitated the atomic structure, such as vacancy agglomeration and motion of pre-existing flaws in the damage layer, inducing time-dependent plastic deformation. As for the holding tests beyond yielding, both stress and strain beneath the indenter were enhanced in comparison to elastic holding, and a severe plastic zone right below contact surface was formed. Under these circumstances, the applied stress was able to meet the requirements for activating creep flow in LiTaO_3_ at room temperature.

**Figure 3:**
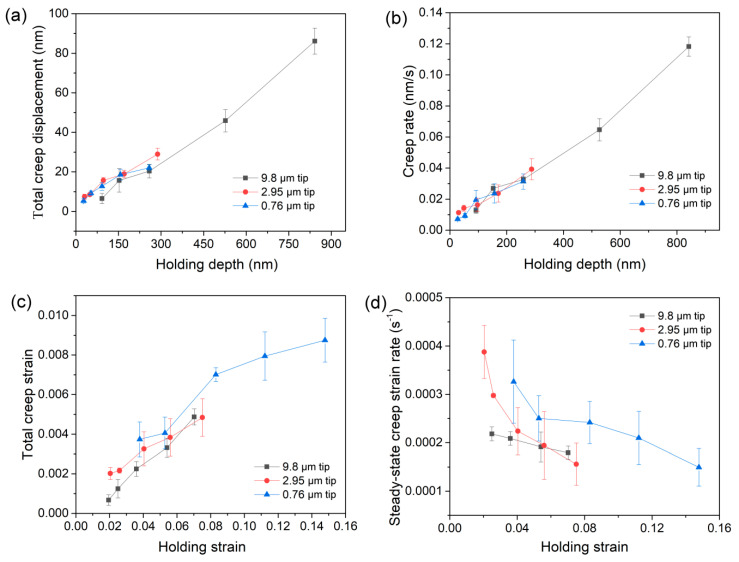
(**a**) Total creep displacement and (**b**) creep rate as a function of holding depth; (**c**) total creep strain; and (**d**) steady-state creep strain rate as a function of holding strain for the three spherical tips.

Figure 3a summarizes the total creep displacements at various holding loads for three spherical tips. Generally, creep displacement tends to increase linearly with increasing holding depth. Creep displacement increased from approximately 5 to 90 nm as holding depth increased from 25 to 850 nm. In comparison, the total creep displacements under spherical tips and Berkovich indenter were similar at holding depths ranging approximately from 100 to 600 nm [16]. Figure 3b exhibits the creep rate in the steady-state stage as a function of holding depth. Creep rate increased from roughly 0.006 to 0.11 nm/s as holding depth increased from 25 to 850 nm. The creep behavior of LiTaO_3_ seems independent of the tip size, and merely correlated with holding depth. There was no discernable distinction between the creep features of elastic and plastic holdings, as was reported in metallic glasses [25]. We could not observe a sudden jump of creep displacement or creep rate as the holding region turned from elastic to plastic in LiTaO_3_.

The indentation size effect on creep deformation needs to be clarified for both apparent and intrinsic reasons. Creep deformation could be described by:

(1) $$\varepsilon_{c}=f\left(\delta ,\varepsilon_{s},T,structure\right)$$

where $\varepsilon_{c}$ is creep strain, δ is applied stress, $\varepsilon_{s}$ is the applied strain on the sample at the beginning of the holding stage, *T* is temperature and *structure* represents all the parameters related to structure. The plastic zone beneath the indenter was gradually expanded with increasing applied load, and creep displacement was in direct proportion to deformed volume *V*. It is reasonable that creep displacement would be larger under a spherical tip with larger size, provided that deformation strains beneath different indenters were similar. Importantly, plastic strain also increased with the application of higher load with the spherical tip, which is the greatest distinction between the spherical tip and the self-similar Berkovich indenter. Therefore, increased deformation strain also provided a great contribution to the increased creep displacement, as we observed. In addition, due to the complicated stress distribution and constraint effect of the elastic surroundings, crack development beneath the indenter would be effectively restricted, resulting in a quasi-plastic deformation zone. In this region, the density and size of micro-cracks and dislocations along different directions could be decreased with increasing holding load. Accordingly, these fertile places provide better atomic mobility for creep flow. Based on the above analysis, the characteristics of creep displacement and creep rate could be insufficient to illustrate the true creep deformation and its correlation with indentation length scale under various holding loads and spherical tips.

Spherical nanoindentation strain could be described as 0.2*α*/*R* [20], where *α* is the contact radius between surface and indenter, and *R* is the radius of the spherical tip. For elastic loading, the contact radius $\alpha =\sqrt{Rh}$ , *h* is the pressed depth. For plastic loading, $\alpha =\sqrt{2Rh_{P}}$ , where *h_p_* is the contact depth, and can be estimated by $h_{p}=h-0.75\frac{p}{S}$ , where *S* is the contact stiffness. Total creep strain under a spherical tip can be calculated by 0.2(*α*-*α*_0_)/*R*, where *a* and *a*_0_ are the contact radii at the beginning and ending of the holding stage, respectively. Figure 3c exhibits the creep strain as a function of holding strain for the three spherical tips. In this case, the effects of holding strain and deformation volume on creep deformation are discernable. As holding strain increases, creep strain increases continuously under each spherical tip. In addition, a sudden enhancement appeared when the holding strain increased from the elastic region to the plastic region, particularly under spherical tips with radii of 0.76 and 2.95 μm. In addition, with respect to the effect of tip size on the creep behavior of LiTaO_3_, it can be concluded that the smaller the deformation volume, the more severe the creep deformation. In comparison, creep strain was about 0.4%, 0.32% and 0.24% under spherical tips with radii of 0.76, 2.95 and 9.8 μm, respectively, when the holding strain was around 5%. As holding strain was increased to ~8%, creep strain under spherical tip with a radius of 0.76 μm approached 7%, which is significantly higher than the creep strains of ~4.5% under the other spherical tips.

The strain rate of steady-state creep is also an important parameter for evaluating the creep resistance of a material [26]. The creep strain rate was calculated by

(2) $$\dot{\varepsilon }=\frac{1}{\sqrt{A}}\frac{d\sqrt{A}}{dt}$$

where *A* is the contact area, which was equal to $2\pi Rh_{p}$ in the plastic region and πRh in the elastic region. The mean value of the creep strain rate in the last 100 s of the holding stage was adopted as the steady-state creep strain rate. Figure 3d shows the correlations between the steady-state creep strain rate and the holding strain for the three spherical tips. A declining tendency was observed for steady-state creep strain rate with increasing holding strain for all of the spherical tips. In comparison, the steady-state creep strain rate was weakly dependent on the holding strain under the spherical tip with a radius of 9.8 μm. It was around 2 × 10^−4^ s^−1^ in the holding strain range from about 2.5% to 7% (the creep strain rate at the minimum holding strain was abandoned due to the giant error bar). Meanwhile, for spherical tips with radii of 2.95 and 0.76 μm, the creep strain rate quickly decreased from 4 × 10^−4^ to 1.5 × 10^−4^ s^−1^ and 3.3 × 10^−4^ to 1.5 × 10^−4^ s^−1^ when holding strain was increased from 2% to 8% for the 2.95 μm tip and 4% to 14% for the 0.76 μm tip, respectively. The declining steady-state creep strain rate suggested that the increased creep strain could mainly be caused by the enhanced creep deformation in the primary creep stage with increasing holding strain. Meanwhile, the tip size effect also appeared whereby creep strain rate was obviously higher under smaller spherical tips at similar holding strains, which was consistent with the creep feature in Figure 3c.

According to the results of both creep strain and strain rate under different-sized spherical tips, it can be concluded that the time-dependent plastic deformation of LiTaO_3_ single crystals led to smaller deformation volumes at the micro/nano scale. In comparison with metals and alloys [27,28,29], effect of size and time on plastic deformation has rarely been investigated in brittle ceramics, due to the difficulty of tracing plastic behaviors by means of macro-mechanical measurements. Several potential reasons could be presented to explain the creep characteristics presented herein under nano/micro contact. The surface or sub-surface damage layers, the thickness of which could range from several hundreds of nanometers to tens of nanometers in LiTaO_3_, might play an important role in creep deformation at the nanoscale. In the damage layer, the atomic arrangement could be severely disturbed, with an amorphous structure even occurring locally, as suggested in the authors’ previous work [7]. Furthermore, structural flaws, i.e., twins and dislocations generated during the grinding process, were difficult to eliminate by means of precise polishing, and were still present in the damage layer. Under similar holding strain conditions, the damage layer occupied a higher proportion of deformation volume under smaller spherical tips. Therefore, we can assume that better atomic motion facilitates creep deformation when adopting a smaller spherical tip. On the other hand, the effect of indentation size on hardness has been widely reported in numerous studies [30,31,32], including with respect to LiTaO_3_ single crystals [16]. It is widely accepted that a higher density of dislocations or other plastic deformation mechanisms beneath the indenter could be facilitated by reducing the nanoindentation depth. Accordingly, instantaneous resistance to plastic deformation, i.e., hardness or strength, could be promoted by the interaction and hindering effects of dislocations or microcracks under nanoindentation. Meanwhile, time-dependent plastic deformation, which is tightly correlated with the pre-existing plastic morphology and structure state at the beginning of the holding stage, could be enhanced by a higher density of structural flaws in the smaller deformation zone beneath the indenter. The role of nanoindentation size in enhancing the creep flow could thus be qualitatively explained, although the intrinsic creep mechanism of LiTaO_3_ remains unknown.

The present creep feature under nanoindentation is close to conventional creep behavior at high temperatures. Therefore, there is merit to estimating strain rate sensitivity (SRS) [33,34,35] in order to reveal the creep mechanism of LiTaO_3_ single crystal and its correlation with the scale of nanoindentation length. The value of the SRS exponent *m* can be evaluated by menas of the following equation:

(3) $$m=\frac{\partial \ln\sigma }{\partial \ln\dot{\varepsilon }}$$

**Figure 4:**
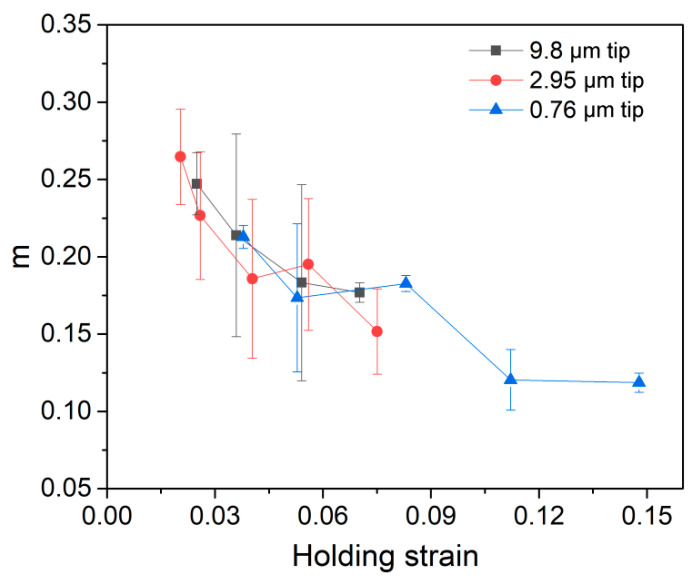
Strain rate sensitivities as a function of holding strain for the three spherical tips.

For a spherical-tip indentation process, the strain rate during the holding stage can be calculated by Equation (2). The creep flow stress σ can be obtained from the mean pressure *P_m_* beneath the indenter via Tabor’s mode, *P_m_ = *3*σ* [36]. In the elastic region under spherical indenters, $P_{m}=\frac{P}{\pi Rh}$ . In the plastic region, the mean pressure can also be defined as hardness, which is $H=\frac{P}{2\pi Rh_{c}}$ for a spherical tip. The SRS value *m* was determined through linear fitting of the log-log correlation between flow stress and strain rate of the steady-state creep curve. Figure 4 exhibits the estimated SRS as a function of holding strain for the three spherical tips, with at least six effective creep curves being adopted for each case in order to ensure the reliability of the result. Obviously, SRS values decreased with increased holding strain for all of the spherical tips. The mean value of SRS was in the range from 0.26 to 0.12 when holding strain was increased from roughly 2% to 15%. Furthermore, the effect of tip size effect on SRS value and its declining tendency can be disregarded. In high-temperature creep flow by uniaxial tension, the creep mechanisms of metals and alloys were empirically tied to SRS values, e.g., 0.1–0.3 for dislocation movement such as dislocation climb and glide; and 0.5 for atomic diffusion along the grain boundary. For the current room-temperature high stress-activated creep deformation under nanoindentation, it is implausible that atomic or vacancy diffusion will occur. For the brittle LiTaO_3_, crack generation and propagation are the predominant manners of plastic or brittle deformation. Meanwhile, in the holding stage, the nanoindentation deformation strain rates were 1 × 10^−4^–4 × 10^−4^ s^−1^, which might be equal to 0.9 × 10^−4^–3.6 × 10^−5^ s^−1^ under uniaxial loading [37]. Under such low strain rates, crack evolution can also be excluded, such that no sudden displacement burst was observed during creep flows in either the elastic or plastic holdings. As described above, the elastic stress might be insufficient to activate the dislocation motion at room temperature. In addition, higher SRS values during elastic holdings could correspond to vacancy agglomeration or the motions of other pre-existing flaws in the damage layer. For the plastic holdings, more fertile places were generated beneath the indenter to meet the requirement of dislocation activation combined with high stress. Dislocation movement might be suggested as the main nanoindentation creep mechanism for LiTaO_3_ single crystals in plastic holdings. The pre-existing dislocation density at the onset of the holding stage would change with nanoindentation depth and strain, explaining the variation in SRS. In addition, the estimated SRS values of the Y-42° plane under spherical tip in plastic holdings were in good agreement with those obtained by Berkovich nanoindentation in the authors’ previous work [16]. This further suggests that the nanoindentation creep mechanism of LiTaO_3_ is independent of indenter type and size. Due to the difficulty of detecting the intrinsic creep deformation manner beneath the indenter, here we qualitatively discuss the potential room-temperature creep mechanisms from the perspective of the plastic mechanism of LiTaO_3_ at the nanoscale and explain the variation of SRS. In the future, the deformation morphologies of the creep deformation suffered as a result of residual nanoindentation will be studied in the cross-section using focus ion beam (FIB) technology and high-resolution transmission electron microscopy (HRTEM).

In the present work, we performed abundant nanoindentation holding tests to reveal the creep behavior in a LiTaO_3_ single crystal, which has rarely been investigated in this soft-brittle ceramic. The novelty of this work and the creep features of LiTaO_3_ can be highlighted as follows:

(1) The nanoindentation size effects, including both pressed depth and applied indenter size, were considered when investigating creep behavior in a LiTaO_3_ single crystal.
(2) Nanoindentation creep displacement and rate during the holding stage were insufficient to represent the creep resistance of a material, because these were comprehensive mechanical responses related to applied strain and/or stress, deformation volume, and structural damage at the beginning of the holding stage.
(3) Nanoindentation creep strain increased with increasing holding strain, while creep strain rate gradually decreased. In addition, the time-dependent plastic deformation of LiTaO_3_ was more pronounced under smaller spherical tips.

## 4. Conclusions

In summary, we investigated the room-temperature creep behavior and its correlation with nanoindentation size effect of a LiTaO_3_ single crystal. By adopting spherical tips with various radii, the effects of holding strain and stress state on creep deformation were carefully evaluated. The tip size effect concerning the deformation zone on creep deformation was also studied. Based on the observed experimental results, several conclusions can be drawn, as below:

(1) Room-temperature creep deformation occurred in LiTaO_3_ by nanoindentation, even under elastic contact. Nanoindentation creep displacement and creep rate increased almost linearly with holding depth, which was superficially uncorrelated with the applied spherical tip size.
(2) Total creep strain was increased with increasing holding strain, while the strain rate of steady-state creep exhibited a declining tendency with increasing holding strain. At a similar holding strain, a nanoindentation size effect appeared, such that creep deformation was more pronounced under a smaller spherical tip.
(3) Strain rate sensitivity (SRS) of creep flow was reduced from 0.26 to 0.12 as holding strain increased from 2% to 15% and was independent of the spherical tip size. Qualitatively, dislocation motion was suggested as the main creep mechanism of LiTaO_3_ single crystal at plastic holdings.

## Supplementary Materials

The following are available online at https://www.mdpi.com/1996-1944/12/24/4213/s1, Figure S1: The statistical distributions of the critical loads at first pop-in under spherical tips of (**a**) 9.8, (**b**) 2.95 and (**c**) 0.76 μm radii, in which critical loads were plotted as a function of measurement. 100 measurements by each tip were performed on LiTaO_3_. This result would be published in the authors’ another work (under submission).
